# From exile to medal: psychosocial wellbeing, belonging, and global solidarity in the Refugee Olympic Team through an ACTM perspective

**DOI:** 10.3389/fpsyg.2026.1799256

**Published:** 2026-07-01

**Authors:** Kadir Caliskan, Sabiha Gizem Engin, Tugce Orsoglu

**Affiliations:** 1 Faculty of Sports Sciences, Bitlis Eren University, Bitlis, Türkiye; 2 Faculty of Sports Sciences, Yozgat Bozok University, Yozgat, Türkiye; 3 Faculty of Sports Sciences, Ankara University, Ankara, Türkiye

**Keywords:** forced displacement, migration health equity, peace and justice, psychosocial wellbeing, social sustainability, SDG-16

## Abstract

**Background:**

This study examines the Refugee Olympic Team (ROT) within the broader context of the modern Olympic Games and the global refugee crisis. While the initiative is frequently framed as a symbol of hope and global solidarity, its broader psychosocial and institutional implications remain underexplored.

**Methods:**

Using an interpretivist qualitative research design, the study conducted qualitative document analysis and thematic analysis of 26 institutional documents, United Nations reports, and international media sources published between 2009 and 2025. The analysis was guided by the Athletic Career Transition Model (ACTM).

**Results:**

The findings show that the ROT provides meaningful symbolic recognition and transition resources by fostering visibility, belonging, hope, and resilience among displaced athletes. However, the analysis also reveals that institutional and media representations frequently construct refugee athletes as exceptional “model migrants,” thereby reinforcing selective inclusion, conditional acceptance, and pressure to perform as idealised symbols of refugee success.

**Conclusion:**

The study highlights the dual character of the Refugee Olympic Team as both a supportive transition space aligned with Sustainable Development Goal 16 and a politically mediated initiative shaped by institutional narratives, structural constraints, and asymmetrical power relations. By integrating ACTM with critical sport-for-development perspectives, this article contributes to scholarship on sport, forced migration, and psychosocial well-being while offering a more explicit psychological interpretation of refugee athletes’ adjustment, coping, and career transition experiences.

## 1 Introduction

Historically, sport has served as an integral instrument in regimes’ endeavours to maintain hegemonic presence and demonstrate power within the international system, as well as to pursue prestige on the world stage (Sisk, 2024). While this historical trend toward the politicisation of sport is observed, it is also seen as a strategic key to building peace, strengthening social cohesion (International Olympic Committee (IOC), n.d.) and even promoting sustainable development (United Nations (UN), 2015). In recent years, sport has additionally been recognised for its role in supporting mental health and psychological wellbeing among populations affected by conflict, and for its growing potential to respond to the needs of refugee athletes seeking hope, continuity, and a sense of belonging. Widely regarded as the climax of the sports arena, the Olympic Games have been recognised as a global event since the dawn of their modern era in 1896, highlighting the unifying power of sports (Galily and Tamir, 2022). Notwithstanding that the Olympics were historically founded upon principles of peace and friendship (Georgiadis, 2009), wars and political conflicts have repeatedly tested the reality of this ideal. Especially during the world wars, some Olympics were cancelled, others suspended, and sometimes political pressures took precedence over sports, restricting participation (Golden, 2011).

After the Arab Spring (Erkkila, 2019), violent conflicts, ethnic tensions and sectarian wars continue, especially in Syria, Iraq, Afghanistan and East Africa (United Nations High Commissioner for Refugees (UNHCR), 2017). The Syrian civil war, which erupted in 2011, has caused the displacement of millions and exacerbated the global refugee crisis (Betts and Collier, 2017). Moreover, the Afghanistan war and ongoing clashes in Iraq have resulted in millions of casualties and forced migration (Seeberg and Eyadat, 2013). Furthermore, due to the Russia–Ukraine war, approximately 4.5 million people came to EU countries from Ukraine as asylum seekers and were placed under temporary protection (Eurostat, 2025). The United Nations also highlights the escalating violence and conflicts in regions such as Ethiopia, Sudan, Myanmar, and the Democratic Republic of the Congo (United Nations High Commissioner for Refugees (UNHCR), 2023). According to the recent reports of the United Nations Refugee Agency, approximately 122 million people have been forcibly displaced worldwide due to persecution, conflict, human rights violations, and disruption of public order, and more than 43 million people continue to live as refugees (United Nations High Commissioner for Refugees (UNHCR), 2025). All these overlapping crises have led to a global refugee crisis, with profound implications for the mental health, psychosocial wellbeing and sense of security of displaced populations, including Olympic-level athletes and inequities among migrant communities. Due to Olympic Charter Rule 41, which states that “any competitor in the Olympic Games must be a national of the country of the NOC entering such competitor” (International Olympic Committee (IOC), 2025), refugee athletes are also unable to represent the host country to which they have fled. In 2015, the International Olympic Committee (IOC) announced the establishment of the Refugee Olympic Team (ROT), describing it as “a symbol of hope and solidarity” (International Olympic Committee (IOC), 2016). Through this initiative, refugee athletes are given the opportunity to compete at the Olympic Games as part of a specially created team (Abd Rahim et al., 2016).

From a psychological perspective, refugee athletes’ experiences can be better understood through Stambulova and Wylleman’s Athletic Career Transition Model (ACTM), which conceptualises athletic transitions as dynamic processes shaped by the interaction among transition demands, available resources, barriers, and coping responses (Stambulova et al., 2021). In the case of refugee athletes, such demands may include forced displacement, trauma, disrupted training pathways, loss of national identity, and uncertainty regarding future belonging; resources may include Olympic Solidarity scholarships, institutional recognition, social support, and access to structured training environments; barriers may involve legal insecurity, exclusion, media pressure, and conditional acceptance; and coping responses may include resilience, identity negotiation, and reliance on social networks. In this study, ACTM is used as a guiding framework to examine refugee athletes’ psychosocial transitions, with particular attention to how demands, resources, barriers, and coping strategies are shaped by forced migration and institutional support. In this sense, ACTM provides a relevant psychological framework for examining how the Refugee Olympic Team may both support and complicate refugee athletes’ adjustment, wellbeing, and long-term career transitions. According to the IOC’s own discourse, the ROT initiative is intended not only to provide refugee athletes with an opportunity to compete at the Olympic Games but also to assist them in continuing their sporting careers and rebuilding their futures through structured forms of athletic and psychosocial support. However, it should not be overlooked that, while the international sports literature pledges to eliminate discriminatory practices, it paradoxically continues to categorise athletes and teams by nationality (Worster, 2022).

In the sports science literature, issues related to migration and refugee athletes are addressed in many studies in sports psychology, and the advantages and disadvantages of refugee status are examined from different perspectives (Agergaard et al., 2023; van Campenhout and Houtum, 2021; Zwahlen et al., 2018). However, with rare exceptions (Erkkila, 2019), the majority of elite athletes in refugee or migrant positions have been observed to face difficulties in adaptation and in developing a sense of belonging (Persad, 2025; Kaya et al., 2021; Jansen and Skey, 2020; Middleton et al., 2020; Burrmann et al., 2017; Schinke et al., 2016; Blodgett et al., 2014). In this context, the ROT project has emerged as an institutional initiative designed to render visible the structural exclusion, loss of belonging and adaptation difficulties experienced by refugee athletes, and to address these challenges through sport in a more systematic way.

Against this conceptual and empirical background, the present study is positioned at the intersection of sport psychology, forced migration studies, and critical analyses of global sport governance. The present study has three specific objectives: first, to analyse ROT’s core psychosocial themes, particularly wellbeing, belonging, and resilience, through the ACTM framework; second, to critically assess IOC institutional representations within the context of the United Nations Sustainable Development Goal 16 (SDG-16: Peace, Justice and Strong Institutions); and third, to propose evidence-based implications for policies and support mechanisms that address refugee athletes’ psychological demands, barriers, and coping needs. These aims can be translated into the following guiding research questions:

1) How are refugee athletes’ psychosocial transition processes represented across institutional and media discourses related to the ROT?
2) In what ways do these representations reflect or challenge the principles of SDG-16 concerning peace, justice, and strong institutions?
3) What implications emerge for the design of support mechanisms that can better respond to refugee athletes’ psychological demands, barriers, and coping needs?

Consistent with research indicating that sport can strengthen psychological resilience, psychosocial wellbeing, and social support mechanisms among forcibly displaced communities, the ROT can be regarded as a distinctive initiative with the potential to promote peace, justice, and global solidarity. In this respect, it also aligns with the Sustainable Development Goals, particularly SDG-16, which emphasises strong institutions and inclusive societies. Highlighting the profound impact of war from the athletes’ perspectives and enhancing global awareness (Bagutti, 2024), this vision, presented by the IOC, serves as a symbol of hope for all refugees worldwide and demonstrates what can be achieved through sport. At the same time, the psychological significance of this initiative lies not only in symbolic visibility but also in the extent to which it supports transition, coping, and long-term adjustment in displaced athletes’ lives.

Critical studies on Sport for Development and Peace underline that such initiatives, while ostensibly designed to support marginalised groups, may operate as paternalistic forms of development stewardship and social control that reproduce existing hierarchies rather than transform them (Darnell and Hayhurst, 2011). Building on this perspective, recent work questions whether refugee participation in the Olympics constitutes genuine empowerment or instead functions of tokenism and “sportswashing” that strengthens institutional legitimacy (Kim, 2024a). In line with these critiques, scholars have raised concerns that, despite its humanitarian rhetoric, the Refugee Olympic Team may inadvertently contribute to the reinforcement of IOC hegemony by channelling global refugee narratives through institutionally controlled platforms, potentially constraining athlete agency and serving, for some observers, as a form of “sportswashing” that benefits the IOC’s public image. Taken together, these perspectives suggest that the ROT can be understood as a paradoxical site where narratives of hope, protection, and global solidarity coexist with critical questions about representation, power, and institutional legitimacy (Bien-Aimé et al., 2024). These tensions make the ROT a particularly important case for examining the intersection of psychological adjustment, institutional support, and mediated representation.

Employing a qualitative interpretivist design based on document analysis, this study examines a purposively selected corpus of institutional reports, United Nations documents, official Olympic materials, and international media sources published between 2009 and 2025. By integrating ACTM with SDG-16 analysis, the study illuminates how forced displacement, Olympic participation, and institutional representation intersect to shape refugee athletes’ psychological wellbeing, transition coping, and long-term adjustment processes (International Olympic Committee (IOC), 2023). Arı (2025) previously examined the Refugee Olympic Team, focusing on Paris 2024 and exclusively on media interviews with the athletes. In contrast, this study centres on the backgrounds and experiences of refugee athletes who participated in the 2016, 2020, and 2024 Olympic Games, emphasising both the challenges they faced and their representations in international media. By combining a psychologically informed career transition lens with a focus on institutional and media narratives across three Olympic cycles, the article aims to offer an integrated perspective on how refugee athletes’ wellbeing and career trajectories are shaped at the intersection of sport, forced migration, and global governance. Through this analytical approach, the study aims to contribute a novel perspective to ongoing scholarly debates at the intersection of sport psychology, forced migration, wellbeing, and global social issues.

## 2 Global crises in the modern Olympic era

The Olympic Games, as an enduring embodiment of friendship, peace, justice, and solidarity, are among the oldest cultural values in human history, dating back thousands of years (Golden, 2011; Spaaij and Burleson, 2016). Regardless of size, wealth, or political system, all nations consider hosting or participating in the Games to be of great value, despite the immense financial burden of organising them (Zirin and Boykoff, 2021), which makes the Olympics the greatest spectacle in the world (Dubinsky, 2023). Hosting the Olympic Games is particularly central to objectives such as enhancing national image, increasing international visibility, promoting tourism, boosting trade, and reinforcing national pride (Ayverdi et al., 2025; Grix, 2013). Beyond these aspects, the Games have evolved into a platform where the underlying psychological, sociological, political, and behavioural influences shaping individual, group, policy, and mass behaviours become manifest (Galily et al., 2024; Lenskyj, 2008; Lenskyj, 2020). In this broader context, it can be understood as disruptive forces that reshape athletes’ career pathways and generate transition demands, a process that is particularly useful to interpret through Stambulova and Wylleman’s Athletic Career Transition Model (ACTM). Within ACTM, sudden disruptions, war-related boycotts or pandemics, may intensify athletic demands, threaten identity continuity, and test coping resources, including institutional support and team solidarity. In this context, the modern Olympic Games have long functioned as a dual space: a global spectacle of unity and a stage for geopolitical tension and reflection of global crises. While founded on ideals of peace and friendship, the Olympic arena has repeatedly served as a mirror of humanitarian and political disruptions.

The 1916, 1940, and 1944 Games were cancelled due to World Wars 1 and 2, and subsequent editions were marked by boycotts reflecting the political tensions of their eras. Despite ‘Rule 50’ of the Olympic Charter, which stipulates that “No kind of demonstration or political, religious, or racial propaganda is permitted in any Olympic sites, venues or other areas” (International Olympic Committee (IOC), 2025), the Games have remained a platform where broader social grievances are expressed. Among the most striking of these crises were the “Black Power Salute” at the 1968 Mexico City Games, denouncing racism and systemic oppression against Black people in the United States (Moore, 2023); the Munich Massacre at the 1972 Games, in which 11 Israeli athletes, coaches, and officials were kidnapped and killed (Carmi and Levental, 2022); the withdrawal of 22 African countries from the 1976 Montreal Games in protest against South Africa’s apartheid regime (Cleveland, 2024); the 1980 boycott of the Moscow Games by 60 countries led by the U.S. following the Soviet invasion of Afghanistan in 1979 (Rice, 2025); and, subsequently, the retaliatory boycott of the 1984 Los Angeles Games by the Eastern Bloc (Petracovschi, 2016). Over time, the governance of Olympic participation has become more tightly regulated by the IOC, such that, under rules introduced following major boycotts, any nation that accepts an IOC invitation but subsequently withdraws may be barred from the subsequent Games (Herguner, 2012).

Finally, in 2020, for the first time in modern Olympic history, the Olympic Games were postponed to 2021 due to the COVID-19 pandemic (Zirin and Boykoff, 2021). This unprecedented decision was taken amid protests from National Olympic Committees, boycott threats, withdrawals by certain national teams (Yuan and Wang, 2022), numerous objections voiced on social media (Sakaki et al., 2022), and opposition from various civil society organisations (Banio, 2021). Announced only a few months prior to the scheduled commencement, the postponement disrupted athletes’ final preparations and had severe economic consequences for the host nation, Japan (de Oliveira et al., 2022). From an ACTM perspective, this pandemic-induced transition illustrates how external barriers such as training restrictions and health-related fears can overwhelm coping resources, while also intensifying psychological strain. The World Athletics Federation (IAAF) stated that the decision was supported by professional sport stakeholders and viewed as necessary to prioritise public health (World Athletics, 2020). This case represented a new type of global crisis, one rooted not in geopolitical conflict but in collective vulnerability, reaffirming the connection between sport, human welfare, and global solidarity (Hoang et al., 2020).

As is well known, the structure of ‘Olympism’, based on peace, friendship, and respect, renders the continuation of the Olympic Games a matter of special significance as one of humanity’s shared cultural heritages (Golden, 2011). This tradition traces back to Antiquity. According to some sources, during the Ancient Olympic Games, wars and political conflicts were suspended under a pact that signified the peace of the gods (hieromania), and this principle was strictly observed (Barbelin, 2004). In ancient Greece, the Olympic Games originated as part of religious rituals aimed at honouring the gods and maintaining harmony through ‘ekecheria’, a sacred truce that ensured peace during the Games. This armistice extended beyond religion, influencing politics and human relations, making the Olympics both a spiritual celebration and a means of fostering peace and unity among the Greek city-states (Bargeliotes, 2009).

Taken together, these historical patterns provide a broader context for understanding the Refugee Olympic Team as a response to disrupted athletic pathways and forced displacement. Regardless of interpretation, politics and political tensions have cast a dark shadow over the Modern Olympic Games. To date, the history of the Modern Olympics has witnessed three cancellations and, due to the global COVID-19 pandemic, one postponement. Beyond the socio-political (Yuan and Wang, 2022) and economic (Suenaga and Takeda, 2021) implications of cancellations or postponements, recent research underscores that such disruptions deeply affect athletes on motivational (Hong and Botwina, 2025; Patatas and Winckler, 2022), emotional (Nowak et al., 2021), psychological (Hooper et al., 2022; Hakansson et al., 2021), and social levels, all of which have significant bearings on their psychosocial wellbeing. Indeed, in a survey conducted by the IOC in May 2020, involving 3,289 athletes, 32% of Olympians reported that the greatest challenge they faced was maintaining their mental health (International Olympic Committee (IOC), 2020). These findings strengthen the argument that sport in times of global crisis extends beyond performance; it becomes a mechanism for resilience, justice, and collective healing through career transition coping - precisely what the ROT aims to provide for displaced athletes within a structured and institutionally mediated environment (Stambulova et al., 2021).

## 3 Shifting paradigms in sport: from winning to participation

**Figure 1:**
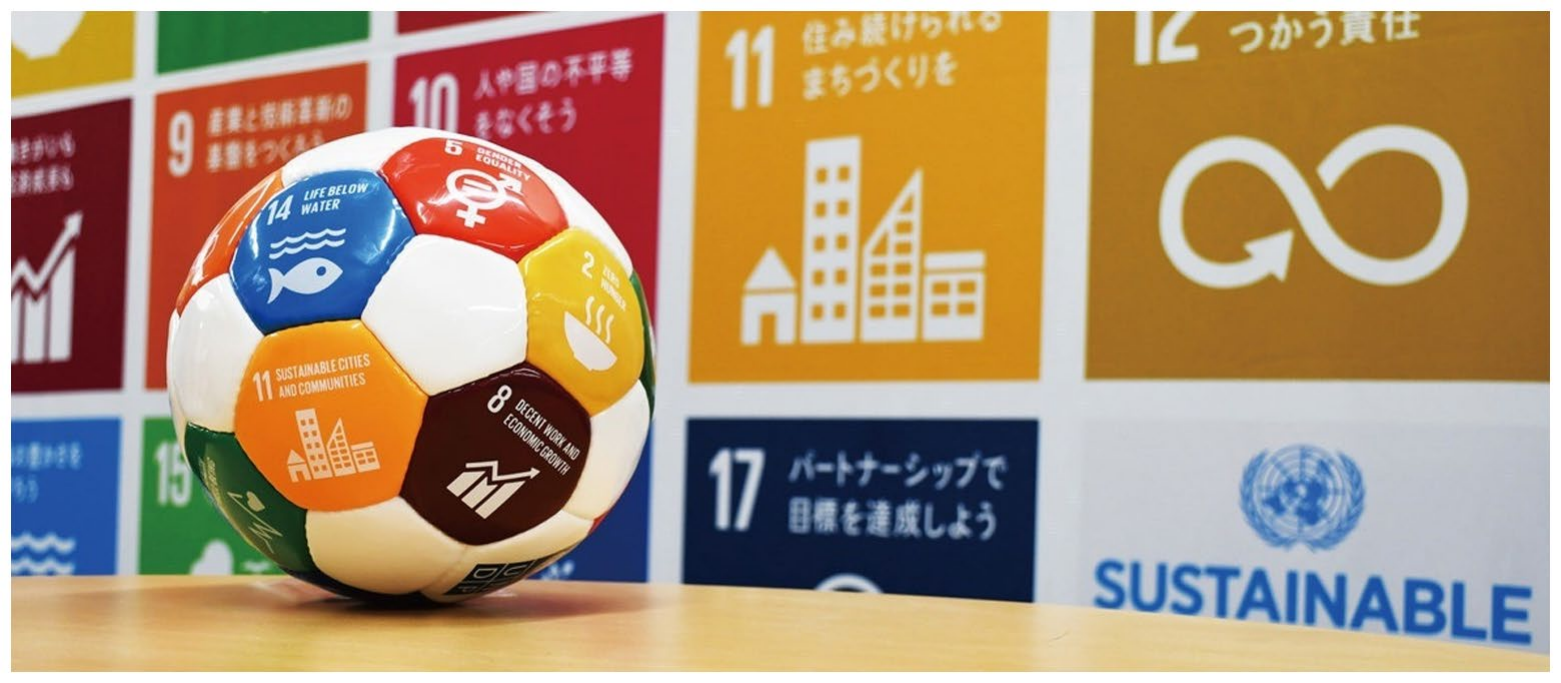
Marking sport for development day (United Nations (UN), 2018).

While historically rooted in amateurism, the modern Olympic paradigm has increasingly integrated the “Sport for Development and Peace” (SDP) framework. From an Athletic Career Transition Model (ACTM) perspective, SDP initiatives may be understood as important resources that help athletes navigate career disruptions by providing structured support systems during periods of instability (Stambulova et al., 2021). In April 2014, the UN and the IOC signed an agreement to promote peace through sport, aligning their missions with the 2030 Agenda for Sustainable Development (United Nations (UN), 2015). UNESCO’s 6th International Conference further defined sport as a functional mechanism for advancing specific objectives, including eight SDG’s (UNESCO, 2017) (Figure 1).

Although sport has been linked to development initiatives, the concept of “Sport for Development and Peace” (SDP) is a relatively recent framework in international development (van Luijk, 2018). The IOC, in line with the United Nations Guiding Principles on Business and Human Rights (UNGPs), has embedded its commitment to human rights in the Olympic Charter, the IOC Code of Ethics, and the IOC Strategic Framework for Human Rights. The IOC was granted Permanent Observer status without a vote on 19 October 2009 (United Nations (UN), 2009), a privilege typically reserved for non-member states and intergovernmental organisations, rarely extended to NGOs (van Luijk, 2018). In 2014, IOC Honorary President Jacques Rogge was appointed as the UN Secretary-General’s Special Representative for Young Refugees and Sport. Later that year, the UN recognised the IOC’s autonomy and the role of sport in advancing education, health, development, and peace. The IOC also regularly participates in the ‘International Day of Peace’, established by the UN General Assembly in 1981 and celebrated annually on September 21 (International Olympic Committee (IOC), n.d.).

These institutional developments can be interpreted as ACTM resources that may facilitate refugee athletes’ career transitions by providing psychosocial support infrastructure, financial stability through Olympic Solidarity scholarships, and opportunities for identity reconstruction during displacement. Additionally, the IOC has implemented and supported numerous SDP programs, such as Sport for Hope, the HIV & AIDS Prevention Program, Olympic Refuge Foundation, Giving is Winning, the Emergency Relief Program, and collaborations with the World Food Programme, UNDP, UN-Habitat, the International Committee of the Red Cross, and UNHCR through the Sport for Refugees initiative (International Olympic Committee (IOC), 2013).

As can be observed, beyond health and wellbeing, the role of sport is also focused on a wide range of societal issues, including education, economic growth, social inequalities, and the promotion of peace and justice. Within the ACTM framework, these SDP mechanisms may function as coping resources that help refugee athletes manage transition demands such as cultural adaptation and trauma recovery, while also navigating barriers including institutional paternalism and conditional belonging (Stambulova et al., 2021). According to Sisk (2024), sport today has become a vehicle of liberal internationalism, envisaged as advancing the attainment of individual rights in areas such as education, health, and gender equality, as well as empowering commonly marginalised groups, including indigenous peoples, traumatised war victims, and individuals with disabilities.

Against this background, the Refugee Olympic Team emerges as a significant SDP-oriented initiative within the broader landscape of athlete support and forced migration. Undoubtedly, sport is regarded as an important tool for refugees to enhance their health, support integration, and improve their wellbeing and sense of belonging (Ley et al., 2021). From this perspective, the ROT may be read as an initiative that provides resources such as team belonging and Olympic visibility, while also helping mitigate barriers linked to national identity loss and fostering coping capacities such as resilience and hope that are important for psychological adjustment during displacement.

## 4 IOC refugee Olympic team

**Figure 2:**
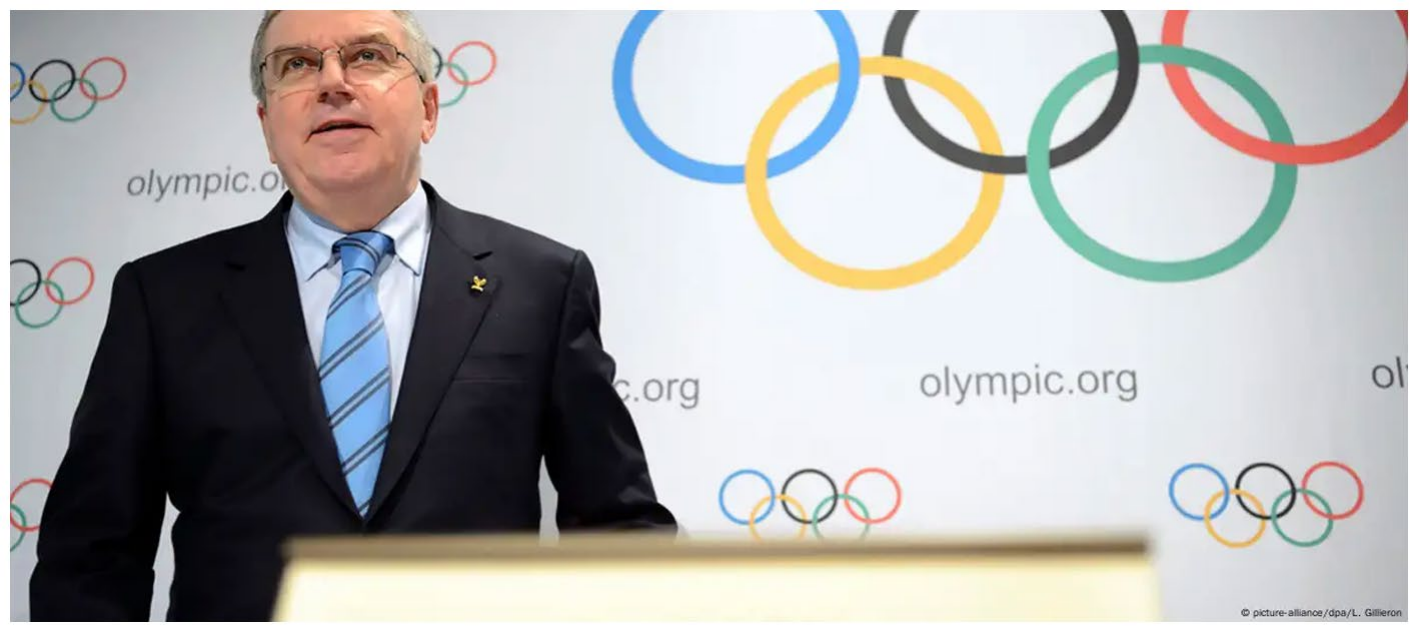
Announcement of first ever Refugee Olympic Team to press (Deutsche Welle (DW), 2016).

According to reports by the United Nations High Commissioner for Refugees (UNHCR), the global refugee crisis, which peaked in 2015, resulted in approximately 70 million people being subjected to forced migration and displacement (United Nations High Commissioner for Refugees (UNHCR), 2015). It has also been reported that more than 1 million refugees seeking asylum in European Union member states have tried to reach here by sea, and approximately 4,000 people have drowned for this cause (Clayton et al., 2015). These displacement-related pressures, such as sudden displacement, family separation, and identity loss, can be understood as severe transition demands that create psychological, social, and practical challenges for refugee athletes. In this context, the Refugee Olympic Team (ROT) was established to meet the needs of athletes whose careers and lives had been disrupted by forced migration. This humanitarian crisis necessitated a new approach consistent with the universal peaceful values of sport. Consequently, in 2016, then-President of the IOC, Thomas Bach (Figure 2), announced the establishment of the first-ever Refugee Olympic Team (ROT) to compete at the Rio Olympic Games, as part of a broader commitment to supporting potential elite athletes affected by the global refugee crisis (International Olympic Committee (IOC), 2016). At the heart of this initiative lies the philosophy of Olympism. As stipulated in the Olympic Charter, the aim of Olympism is to position sport as a means of promoting a peaceful society concerned with the preservation of human dignity (International Olympic Committee (IOC), n.d.).

Following this announcement, the IOC requested National Olympic Committees (NOCs) worldwide to identify refugee athletes with the potential to qualify for the Rio 2016 Olympic Games. Initially, 43 promising candidates were identified, and after careful evaluation, ten refugee athletes were selected to form the first IOC Refugee Olympic Team. This step enabled refugee athletes who had been forced to flee their countries due to war and conflict, yet were unable to compete under the banner of a new nation, to appear on the Olympic stage. In doing so, it embodied universal principles of human rights and inclusivity while serving as a powerful symbol of hope for refugees worldwide (Giffin et al., 2024). The creation of the Refugee Olympic Team can therefore be interpreted as an important career transition intervention within the broader fields of global sports diplomacy and humanitarian action (de Almeida and Rubio, 2023; Abdi et al., 2019). Furthermore, the project represents a political and structural transformation aimed at addressing humanitarian needs, expanding the Olympic Movement’s inclusivity and peace-building mission (Burdsey et al., 2023). In this sense, the initiative may also be understood as a practical expression of the Olympic Movement’s claim to support inclusion and peace-building in conditions of displacement.

In identifying and supporting athletes for the Refugee Olympic Team, the IOC collaborated with the UNHCR to develop a specific selection procedure targeting athletes with officially recognised refugee status in line with international standards (International Olympic Committee (IOC), 2022). This method was designed to expand training opportunities for athletes, oversee sporting evaluation processes, and establish psychosocial support mechanisms (Scwartzkopff, 2022). From an ACTM perspective, this process reflects a balance between transition demands and available resources. In this way, refugee athletes were enabled not only to reach a competitive sporting level but also to receive broader social support (Giffin et al., 2024). Athletes’ performances were evaluated within the framework of international federation criteria, with active involvement of coaches, health professionals, and psychologists throughout the process (Giffin et al., 2024). Moreover, the selection process took into account not only the athletes’ physical capacities but also their psychological resilience and potential for social adaptation (Braniste, 2022). According to Carlo Bagutti, the chief medical officer of the Refugee Olympic Team, the reason for this lies in the fact that each athlete within the team has undergone an exceptionally challenging life journey, marked by profound traumatic experiences, while also carrying particular concerns for family members and loved ones who remain in precarious and vulnerable circumstances. In addition to this already heavy emotional burden, some athletes become targets of ‘smear campaigns’ originating from their former countries. Although the athletes themselves are frequently subjected to exploitation, persecution, or even treated as criminals, they are often unjustly accused by state-controlled media of betraying national values or of having misused the support previously provided to them by their former governments (Bagutti, 2024). Taken together, these factors indicate that the ROT selection process addressed not only athletic readiness but also psychosocial vulnerability and the wider social conditions surrounding displacement. Through this multidimensional approach, the initiative sought to ensure that athletes joining the team could successfully adapt to the Olympics.

In 2024, the British Journal of Sports Medicine (BMJS) published an issue featuring Dr. Carlo Bagutti’s experiences as a service spotlight. Bagutti’s (Bagutti, 2024) reflections embody a profound sense of humanity:

Stress may impact emotional balance, quality of sleep, concentration and recovery, and ultimately the quality of performance. Supporting these courageous athletes in navigating these challenges is both deeply rewarding and profoundly inspiring. For all these reasons, it has been a privilege and honour to serve as the Chief Medical Officer of the EOR delegation for the Rio 2016, Tokyo 2020 and Paris 2024.

These reflections illustrate how the ROT initiative is not only about enabling participation at the elite level but also about protecting and promoting refugee athletes’ mental health and psychological wellbeing throughout their Olympic journey. Alongside these measures, and in line with IOC policies, the visibility of the Refugee Olympic Team has been strategically enhanced through media coverage to draw global attention to the refugee crisis and raise awareness within the international community (Michelini, 2021a). This visibility may serve two ACTM-related functions: it can provide external validation of coping resources that strengthen belonging, and it can increase global awareness of refugee athletes’ circumstances. Although the IOC largely refrained from taking a significant stance on major human rights issues, particularly from the 1936 Berlin Nazi Olympics to the anti-apartheid boycotts of the 1960s (Cottrell and Nelson, 2014), the establishment of the Refugee Olympic Team can be interpreted as a conscious sensitivity to global humanitarian crises and a pioneering career transition support model for displaced elite athletes. At the same time, this initiative may be viewed as a limited but meaningful departure from earlier periods in which the IOC adopted a more restrained position on major political and human rights crises.

## 5 Methodology

### 5.1 Research design

This study was conducted using a qualitative research design grounded in the interpretivist paradigm, aiming to provide a contextual understanding of the Refugee Olympic Team’s role as a symbol of peace, justice, psychosocial wellbeing, and global solidarity (Creswell and Poth, 2018). The interpretivist approach was selected because it enables the examination of meanings embedded in institutional texts, media narratives, and policy documents, rather than limiting the analysis to descriptive content alone.

### 5.2 Data sources

As presented in Table 1, the study consists of a corpus of 26 institutional publications, international organization reports, official Olympic documents, and selected international media texts published between 2009 and 2025. These documents were obtained from publicly accessible databases of organisations such as the United Nations, the International Olympic Committee, the United Nations High Commissioner for Refugees (UNHCR), the World Health Organization (WHO), UNESCO, and other relevant institutional platforms, alongside international media outlets.

**Table 1:** Key documents used in the study.

Year	Source/Report name	Content
2009	UN	General Assembly Report
2013	IOC	IOC Factsheet
2015	UN Transforming our world	UN Report
2015	United Nations High Commissioner for Refugees	Global Trends Report
2016	United Nations High Commissioner for Refugees	Global Trend Report
2016	IOC	ROT Representation
2016	BBC	News - Interview
2016	Deutsche Welle	News
2016	World Olympians Association	WOA News
2016	IOC	ROT 2016 Representation
2017	UNESCO, Kazan Plan	UNESCO Report
2018	UN	Website
2020	World Athletics	Press Release
2020	IOC, Athlete365	Survey Findings
2020	IOC	ROT 2020 Representation
2020	World Health Organization	WHO News
2021	Info Migrants	Int. Off. News Platform
2022	IOC	IOC News
2023	United Nations High Commissioner for Refugees	Annual Report
2024	United Nations High Commissioner for Refugees	Annual Report
2024	Carlo Bagutti, ROT Chief Medical Officer	Medical Report
2024	IOC	ROT 2024 Representation
2024	IOC	IOC News
2024	United Nations High Commissioner for Refugees	UNHCR News
2024	International Rescue Committee	IRC News
2025	Eurostat, Statistics	Official Statistics
2025	IOC	Olympic Charter
2025	IOC	IOC News
2025	IOC	IOC News
n.d.	IOC	Website

Document selection adhered to purposive sampling criteria, specifically targeting sources that addressed the Refugee Olympic Team, refugee policies in sport, and institutional perspectives on social inclusion and peace-building. Only publicly accessible documents produced by recognised international organisations or reputable media outlets were included to ensure credibility and traceability of the data sources. In addition, the corpus was organised to capture both institutional and media representations across different Olympic cycles, allowing the study to compare how the ROT was framed over time.

### 5.3 Data collection and analysis

To examine the Refugee Olympic Team’s role within the United Nations Sustainable Development Goal 16 (SDG-16: Peace, Justice and Strong Institutions) framework, qualitative document analysis was adopted as the primary data collection strategy, systematically transforming secondary data into meaningful insights (Bowen, 2009). Thematically coded meaning units were used to explore themes of peace, justice, global solidarity, belonging, hope, and psychosocial wellbeing (Mayring, 2015). The analytical strategy was also informed by the psychological transition perspective introduced in the introduction, particularly the Athletic Career Transition Model (ACTM), which guided the interpretation of athletes’ demands, resources, barriers, and coping processes across the dataset (Stambulova et al., 2021).

The analysis proceeded in two stages. *Stage 1 - Document Review and Data Extraction:* At this stage, documents systematically reviewed IOC reports, UNHCR documents, Olympic Games official open-access materials, and diverse international media texts and visuals to identify Refugee Olympic Team policies, practices, outcomes, and social sustainability indicators (e.g., psychosocial wellbeing, inclusivity, inequality reduction, fostering belonging). During this stage, relevant statements, narratives, and representations of refugee athletes were extracted as meaning units and organised for further coding. *Stage 2 Thematic Coding and Deductive Content Analysis:* Stage 2 subjected the preliminary findings to a deductive content analysis (Elo and Kyngäs, 2008), categorising them into predefined themes. The coding framework was developed in relation to the theoretical perspective of SDG-16 and the psychosocial dimensions frequently associated with refugee integration in sport and the ACTM-based reading of transition experiences. Accordingly, the analysis focused on recurring expressions and narratives connected to four key interpretive dimensions:

- psychosocial wellbeing
- hope
- sense of belonging
- peace, justice, and institutional support (SDG-16)

These themes provided the structure for coding across the dataset, enabling the identification of patterns in how the ROT is framed in institutional and media discourse. The coding process identified recurring expressions and patterns related to social sustainability within the dataset, linking themes to the theoretical framework of SDG 16 and Olympic solidarity. To enhance research reliability, data sources were predominantly selected from official and institutional documents. Findings were validated through consistency checks across document sections and years, thereby strengthening the study’s internal validity. Furthermore, triangulation across institutional documents and media representations allowed cross-verification of emerging themes, increasing the robustness of the interpretive analysis. This aligns with methodological coherence in qualitative sport psychology, ensuring alignment from interpretivist ontology to thematic findings (Poucher et al., 2020).

## 6 Findings and discussion

### 6.1 2016 Rio Olympics (Brazil)

**Figure 3:**
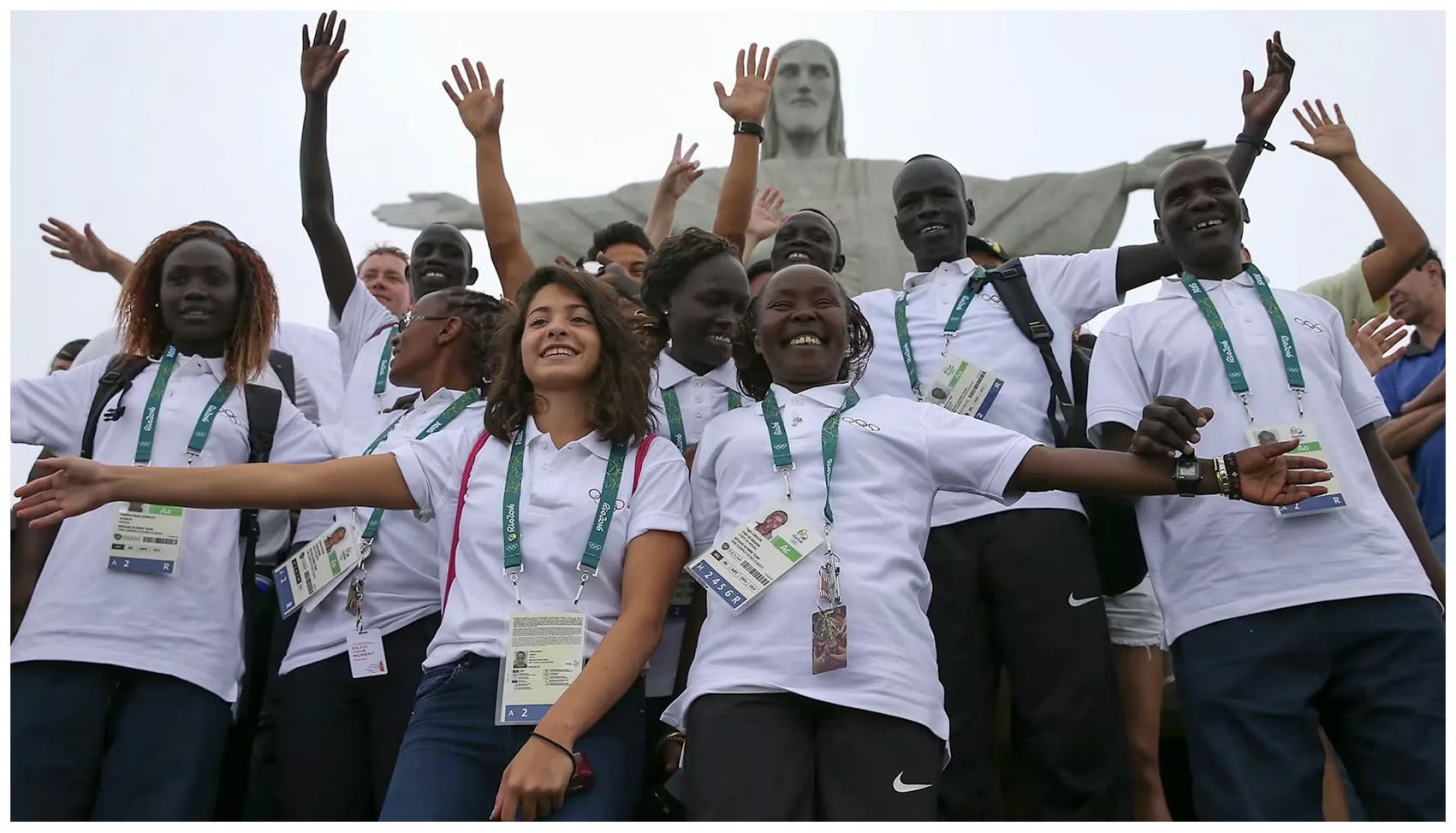
First ever refugee Olympic team in Brazil for 2016 Rio Olympics (International Olympic Committee (IOC), 2016).

The first Refugee Olympic Team established by the IOC at the 2016 Rio Olympics had a total of 10 athletes (Figure 3), six in athletics and two each in swimming and judo, and only three different disciplines (International Olympic Committee (IOC), 2016). The team’s chief of mission was Kenya’s former long-distance athlete Tegla Loroupe, who broke the world marathon record in 1998 as the first African woman athlete (World Olympians Association (WOA), 2016). Loroupe has also been recognised internationally for her advocacy for women’s rights, education, and peace, and was later honoured by the United Nations as Person of the Year. During all formal representations of the team, such as medal presentations, the Olympic flag flew and the Olympic anthem played.

The list of these athletes is given in Table 2 (International Olympic Committee (IOC), 2016).

**Table 2:** The first Refugee Olympic Team to take part in the 2016 Rio Olympics.

Sport	Name	Gender	Origin	Host NOC
Athletics	Anjaline N. Lohalith	W	South Sudan	KEN
Athletics	James N. Chiengjiek	M	South Sudan	KEN
Athletics	Paulo A. Lokoro	M	South Sudan	KEN
Athletics	Rose N. Lokonyen	W	South Sudan	KEN
Athletics	Yiech P. Biel	M	South Sudan	KEN
Athletics	Yonas Kinde	M	Ethiopia	LUX
Judo	Popole Misenga	M	Congo	BRA
Judo	Yolande B. Mabika	W	Congo	BRA
Swimming	Rami Anis	M	Syria	BEL
Swimming	Yusra Mardini	W	Syria	GER

At the end of the Games, none of the athletes succeeded in winning a medal. However, the words of the then IOC President, Thomas Bach, clearly illustrate the primary mission of the team (International Olympic Committee (IOC), 2016):

The Refugee Olympic athletes were some of the stars of these Olympic Games. They were stars in a way that they demonstrated the best of human beings; they demonstrated determination; they demonstrated what you can achieve if you want to. They also demonstrated that they are not simply refugees but that they are human beings.

Such recognition not only validated their athletic performances but also contributed to restoring their sense of dignity, hope and psychological wellbeing on the world stage. From an ACTM perspective, the Rio 2016 team represented an early transition space in which refugee athletes faced intense demands (displacement histories, limited preparation, uncertainty) while gaining symbolic resources (Olympic visibility, team recognition, institutional support) that helped them begin rebuilding their identities and sense of belonging. Although the Refugee Olympic Team did not win any medals at Rio 2016, this outcome should not be interpreted as a failure. Rather, the Rio case shows that success cannot be reduced to medals alone; it illustrates how career transition support can foster adjustment, resilience, and psychological continuity under forced-migration conditions.

### 6.2 2020 Tokyo Olympics (Japan)

**Figure 4:**
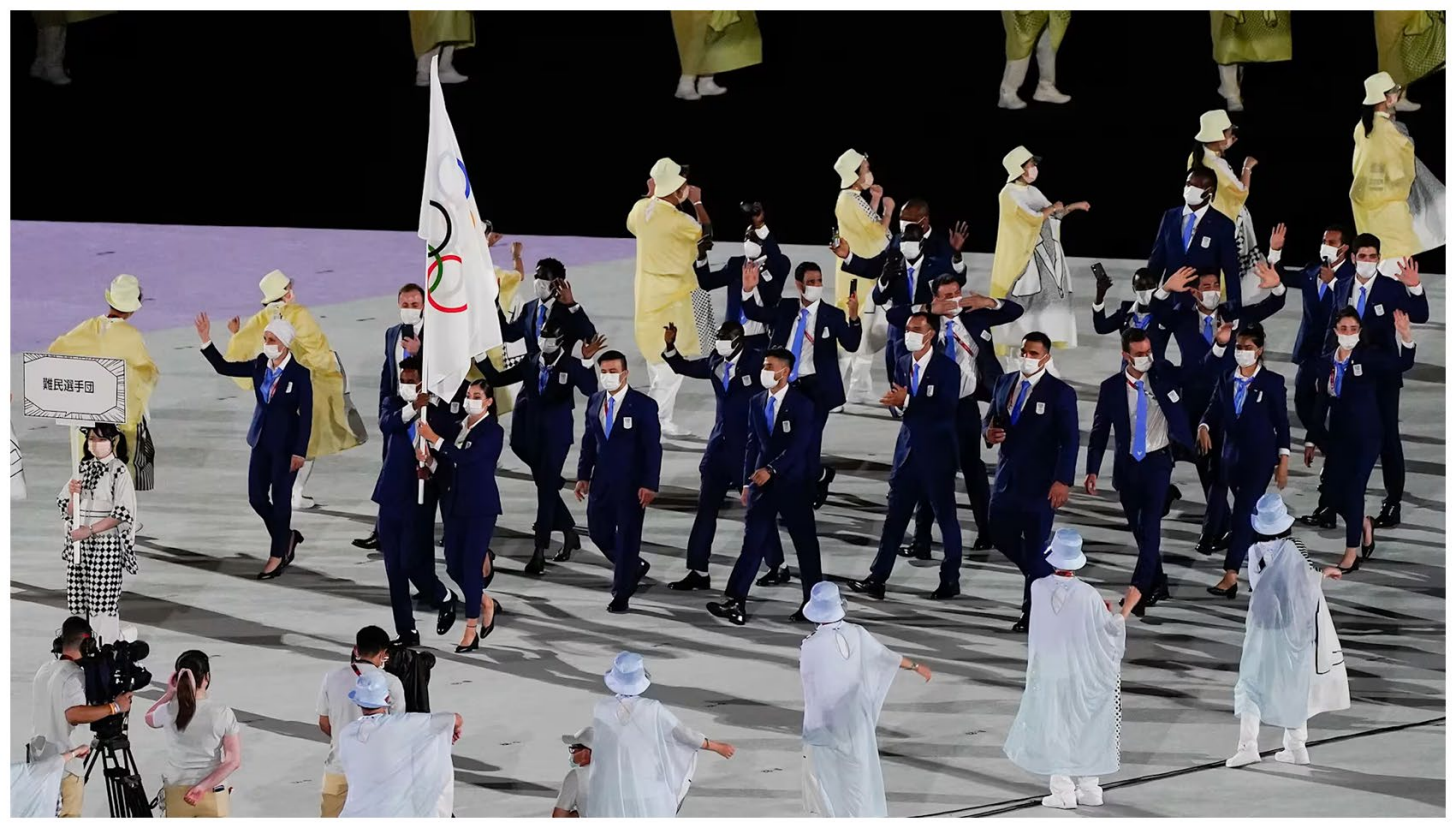
Refugee Olympic Team in opening ceremony at Tokyo Olympics (International Olympic Committee (IOC), 2020).

In 2018, the IOC announced that the Refugee Olympic Team would be continued at next Games. The abbreviation of the team was changed from ROT to EOR, which was expressed as “*Équipe olympique des réfugiés*” in French. Through the Olympic Solidarity scholarships, 56 athletes from 21 host countries (Australia, Austria, Belgium, Brazil, Canada, Croatia, Egypt, France, Germany, Israel, Jordan, Kenya, Luxembourg, Portugal, the Netherlands, New Zealand, Trinidad and Tobago, Türkiye, Sweden, Switzerland, and the United Kingdom) received financial support to prepare for the Games. Selections were made among these fellows based on a number of criteria, including sporting performance and refugee status verified by UNHCR (International Olympic Committee (IOC), 2020). At the end of the selection stages a total of 29 athletes from 11 countries were selected, and they competed in 12 different sports including seven in athletics, six in judo, three in taekwondo, two in cycling, two in boxing, two in karate, two in swimming, one in wrestling, one in badminton, one in weightlifting, one in shooting, and one in canoeing (International Olympic Committee (IOC), 2016). The team’s mission chief was Tegla Loroupe, who had previously carried out this mission at Rio 2016 (Figure 4; Mackay, 2021).

Details about these athletes are given in Table 3 (International Olympic Committee (IOC), 2020).

**Table 3:** Refugee Olympic Team to take part in the 2020 Tokyo Olympics.

Sport	Name	Gender	Origin	Host NOC
Athletics	Anjaline N. Lohalith	W	South Sudan	KEN
Athletics	Dorian Keletela	M	Congo	POR
Athletics	Jamal A. E. Mohammed	M	Sudan	ISR
Athletics	James N. Chiengjiek	M	South Sudan	KEN
Athletics	Paulo A. Lokoro	M	South Sudan	KEN
Athletics	Rose N. Lokonyen	W	South Sudan	KEN
Athletics	Tachlowini Gabriyesos	M	Eritrea	ISR
Badminton	Arem Mahmoud	M	Syria	NED
Boxing	Eldric S. Rodriguez	M	Venezuela	TTO
Boxing	Wessam Salamana	M	Syria	GER
Canoe	Saeid Fazloula	M	Iran	GER
Cycling	Ahmad B. Wais	M	Syria	SUI
Cycling	Masomah A. Zada	W	Afghanistan	FRA
Judo	Ahmad Alikaj	M	Syria	GER
Judo	Javad Mahjoub	M	Iran	CAN
Judo	Muna Dahouk	W	Syria	NED
Judo	Nigara Shaheen	W	Afghanistan	RUS
Judo	Popole Misenga	M	Dr Congo	BRA
Judo	Sanda Aldass	W	Syria	NED
Karate	Hamoon Derafshipour	M	Iran	CAN
Karate	Wael Shueb	M	Syria	GER
Shooting	Luna Solomon	W	Eritrea	SUI
Swimming	Alaa Maso	M	Syria	GER
Swimming	Yusra Mardini	W	Syria	GER
Taekwondo	Abdullah Sediqi	M	Afghanistan	BEL
Taekwondo	Dina P. Langeroudi	W	Iran	NED
Taekwondo	Kimia Alizadeh	W	Iran	GER
Weightlifting	Cyrille Tchatchet II	M	Cameroon	GBR
Wrestling	Aker A. Obaidi	M	Iraq	AUT

On 11 March 2020, due to the continuation of an acute respiratory illness with global impact and the uncontrolled spread of the virus in several countries, the World Health Organization (WHO) declared a pandemic (World Health Organization (WHO), 2020). Although only 5 months remained until the Games, the WHO’s position was taken into account; the IOC announced the postponement of the Tokyo 2020 Olympic and Paralympic Games to 2021 (Goh and Kano, 2020). However, the Tokyo Olympics were held a year later, albeit with a delay in controversy. Although refugees did not win medals at these games, as in the Rio 2016 Olympics, they have brought the issue of migration back to the agenda as their visibility in the media has increased (Basnet, 2024). Therefore, it would be more accurate to evaluate the success criterion in the context of the team’s vision and mission, not the number of medals, including its contribution to enhancing awareness of refugees’ lived experiences and supporting their sense of dignity, hope and psychological wellbeing.

Within the ACTM framework, the Tokyo postponement can also be understood as a compounded career transition: refugee athletes were already managing forced-displacement demands, and the pandemic added a new layer of barriers such as disrupted training, uncertainty, and heightened mental strain. At the same time, Olympic Solidarity scholarships functioned as transition resources, while the postponement itself tested coping capacities and made psychological support even more necessary for adjustment and continuity.

### 6.3 2024 Paris Olympics (France)

At the 2024 Paris Olympic Games, the Refugee Olympic Team was represented by a total of 37 athletes from 11 different countries across 12 disciplines. These included 8 athletes in athletics, 6 in judo, 5 in taekwondo, 4 in canoeing, 2 each in shooting, cycling, boxing, wrestling, weightlifting, and karate, and 1 each in swimming, badminton, and breakdancing (International Olympic Committee (IOC), 2024). Gender and regions were also considered for balanced representation. In the meantime, the Paris 2024 Olympic Games marked a significant milestone in history as the first edition to feature an equal number of male and female athletes (International Olympic Committee (IOC), 2025).

**Figure 5:**
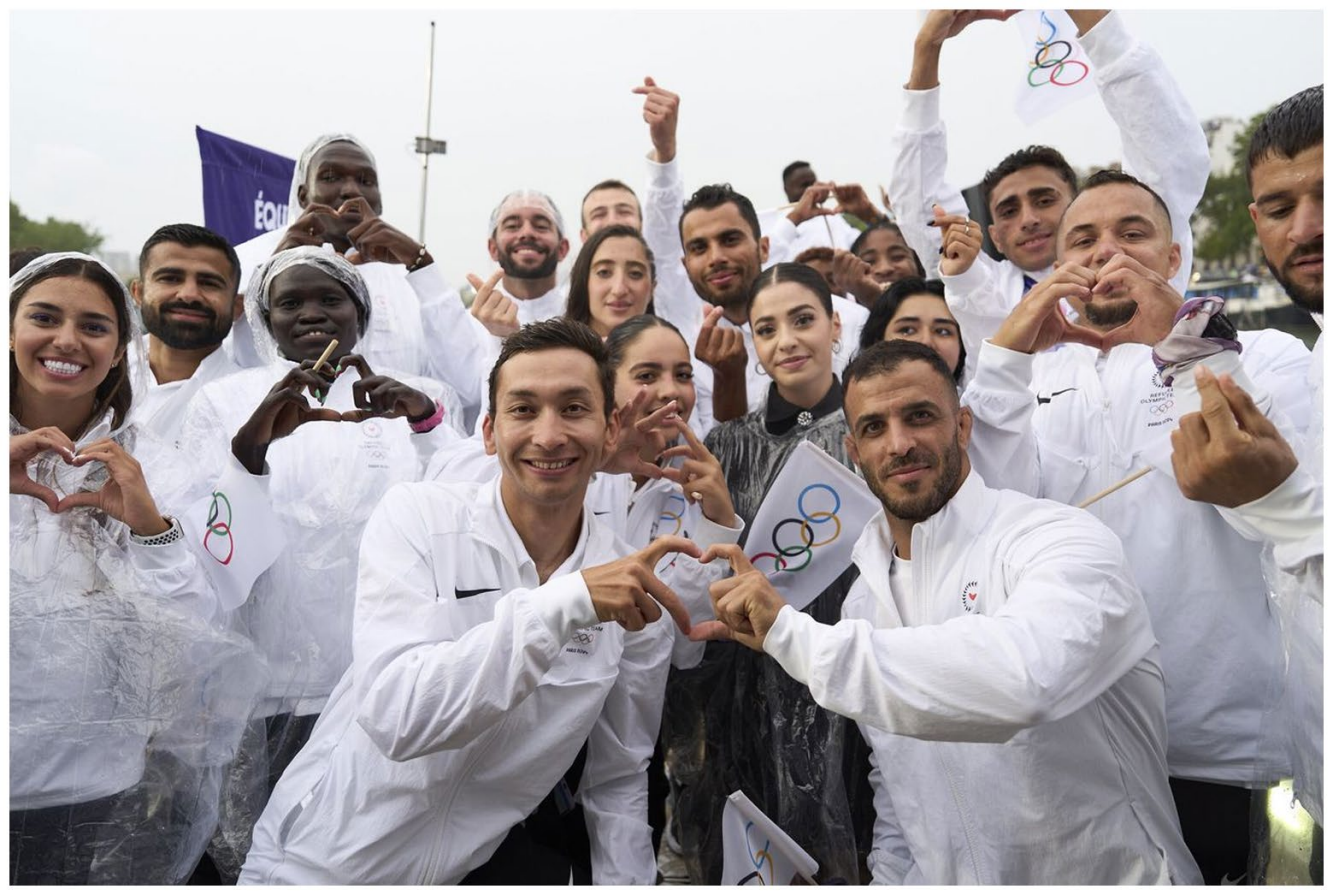
Refugee team in opening ceremony at 2024 Paris Olympics (Bagutti, 2024).

Team leadership was undertaken by Masomah Ali Zada, a former member of the 2020 Refugee Olympic Team (Figure 5). The majority of the athletes on the team were supported through the Refugee Athlete Scholarship Programme, funded by Olympic Solidarity.

Details about these athletes are given in Table 4 (International Olympic Committee (IOC), 2024).

**Table 4:** Refugee Olympic Team to take part in the 2024 Paris Olympics.

Sport	Name	Gender	Origin	Host NOC
Athletics	Dominic Lobalu	M	South Sudan	SUI
Athletics	Dorian Keletela	M	Dr Congo	FRA
Athletics	Farida Abaroge	W	Ethiopia	FRA
Athletics	Jamal A. E. Mohammed	M	Sudan	ISR
Athletics	Mohammad A. Alsalami	M	Syria	GER
Athletics	Musa Suliman	M	Sudan	SUI
Athletics	Perina Lokure	W	South Sudan	KEN
Athletics	Tachlowini Gabriyesos	M	Eritrea	ISR
Boxing	Cindy Ngamba	W	Cameroon	GBR
Badminton	Dorsa Yavarivafa	W	Iran	GBR
Boxing	Omid Ahmadisafa	M	Iran	GER
Breaking	Manizha Talash	W	Afghanistan	ESP
Canoeing	Amir Rezanejad	M	Iran	GER
Canoeing	Fernando Jorge	M	Cuba	USA
Canoeing	Saeid Fazloula	M	Iran	GER
Canoeing	Saman Soltani	W	Iran	AUT
Cycling	Amir Ansari	M	Afghanistan	SWE
Cycling	Eyeru T. Gebru	M	Ethiopia	FRA
Judo	Adnan Khankan	M	Syria	GER
Judo	Mahboubeh Barbari	W	Iran	GER
Judo	Mohammad Rashnonezhad	M	Iran	NED
Judo	Muna Dahouk	W	Syria	NED
Judo	Nigara Shaheen	W	Afghanistan	CAN
Judo	Sibghatullah Arab	M	Afghanistan	GER
Shooting	Francisco E. C. Nieves	M	Venezuela	MEX
Shooting	Luna Solomon	W	Eritrea	SUI
Swimming	Alaa Maso	M	Syria	GER
Swimming	Matin Balsini	M	Iran	GBR
Taekwondo	Dina Pouryounes	W	Iran	IRI
Taekwondo	Farzad Mansouri	M	Afghanistan	AFG
Taekwondo	Hadi Tiran	M	Iran	ITA
Taekwondo	Kasra Mehdipournejad	M	Iran	IRI
Taekwondo	Yahya Al-Ghotany	M	Syria	JOR
Weightlifting	Ramiro M. Romero	M	Cuba	CUB
Weightlifting	Yekta Jamali	W	Iran	IRI
Wrestling	Iman Mahdavi	M	Iran	IRI
Wrestling	Jamal Valizadeh	M	Iran	IRI

**Figure 6:**
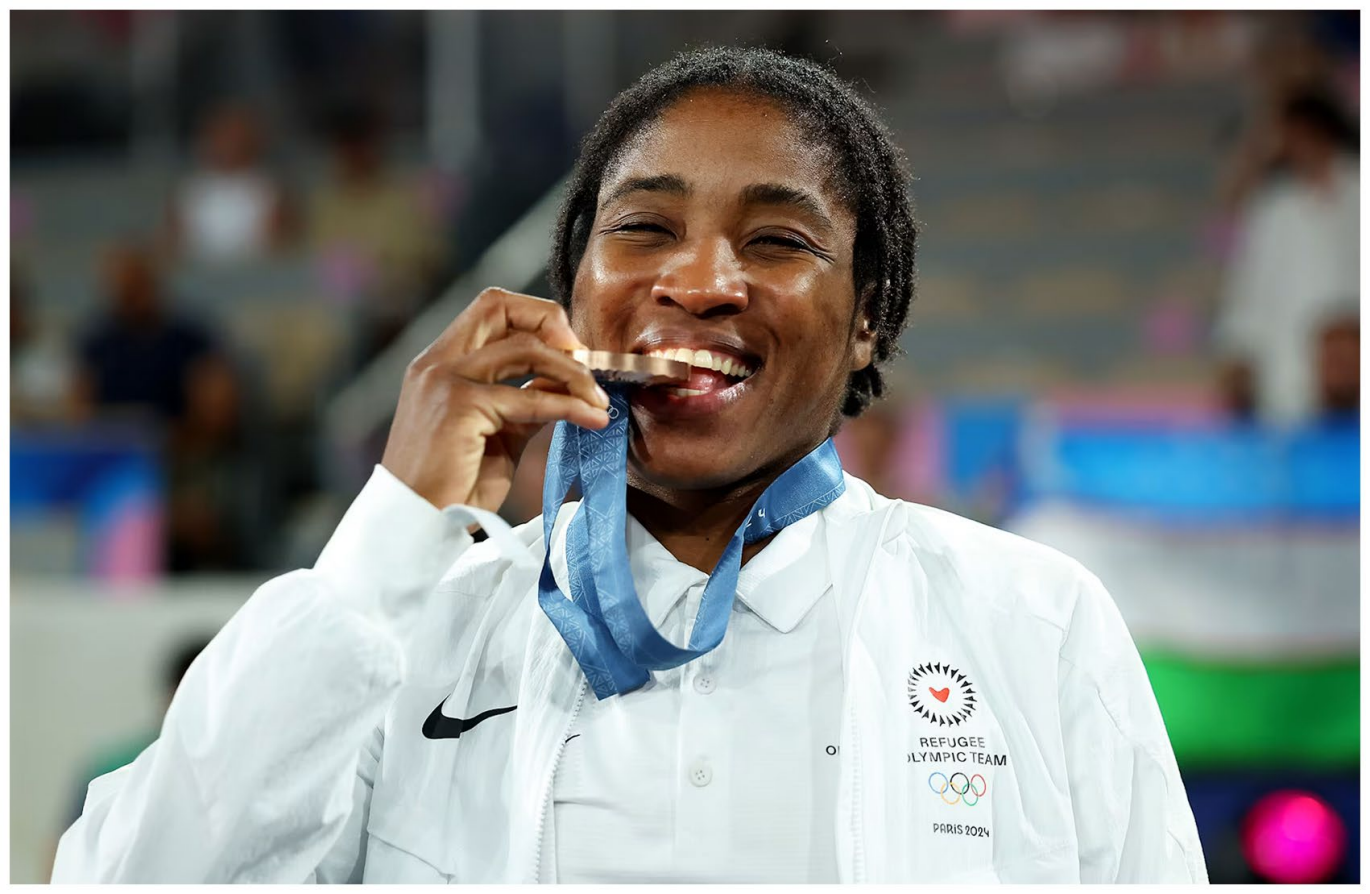
First medal for EOR by Cindy Ngamba at 2024 Paris Olympics (International Olympic Committee (IOC), 2024).

At the 2024 Paris Olympics, the success that had been dreamed of for so long was finally achieved. Cameroonian-born Cindy Ngamba secured a bronze medal in boxing, marking a historic first in Olympics and symbolising a beacon of hope and belonging with important implications for psychological health of displaced athletes who identify with her story for potential future members of this team across the globe (Figure 6) (International Olympic Committee (IOC), 2024).

Jojo Ferris, Head of the Olympic Refuge Foundation (ORF), described this historic achievement with the following words (The UN Refugee Agency (UNHCR), 2024):

Cindy reminds us what refugees can and do achieve, how they thrive if they are given the opportunity and what a positive contribution they make to communities around the world. This is a huge moment for Cindy, the IOC Refugee Olympic Team, and 120 million people across the world that have been forced to flee their homes.

Ngamba’s medal should be read as both an outcome of successful adaptation and a resource for other displaced athletes, because it combines visibility, affirmation, and hope with the possibility of identity reconstruction. However, the same achievement also reflects the model’s barrier dimension, as exceptional success can increase pressure, conditional acceptance, and the expectation that refugee athletes must continuously prove their worth.

### 6.4 Refugee athletes’ voices and lived experiences

To ground institutional claims in subjective realities and examine psychological wellbeing through athletes’ own experiences of trauma, resilience, and identity negotiation, this section centres first-person narratives from a few Refugee Olympic Team athletes. These accounts illuminate ROT’s dual psychosocial impact; while fostering belonging and hope, they simultaneously expose vulnerabilities to conditional acceptance, deportation fears, and persistent trauma. These themes resonate fully with SDG 16’s peace and justice imperatives through reconstituted solidarity. They also align closely with ACTM, as they illustrate how athletes actively navigate the interplay of transition demands, institutional resources, systemic barriers, and coping strategies.

Regarding the broader impact of Cindy Ngamba’s success, Shabnam Salahshoor, an Afghan refugee who participated in the ORF’s ‘Terrains d’Avenir’ programme, offered a poignant perspective. Through this program, she has been endeavouring to rebuild her life. In this context, Salahshoor completed football coaching training and expressed that she felt as if the medal belonged to all refugees (International Olympic Committee (IOC), 2025):

I felt she was me… You’ve got this, Cindy! You’ve got this!

The phrase ‘I felt it was me’ may have perfectly captured EOR’s vision in its shortest form. In this context, the role of ‘Olympic Education’ programs in promoting social cohesion and fostering mental health and psychological wellbeing should not be overlooked (Örsoğlu and Yıldıran, 2025). ACTM helps explain why this moment matters: the medal served as a symbolic coping resource, enabling identity connection, shared hope, and emotional validation amid displacement-related strain.

Nevertheless, Ngamba’s historic medal also exemplifies the psychosocial ‘double-edged sword’ of exceptional success; while it symbolises profound hope and elevates collective dignity for millions (United Nations High Commissioner for Refugees (UNHCR), 2025), it simultaneously generates conditional recognition and new performance pressures. As “model migrants,” such athletes risk becoming tokenised symbols whose value hinges on performance, potentially exacerbating anxiety over future failures or deportation fears. This paradox aligns with SDG 16’s justice imperative, highlighting sport’s transformative potential alongside risks of precarious social acceptance (Burdsey et al., 2023). In ACTM terms, this is the point where resources and barriers coexist: the same visibility that supports adjustment can also intensify pressure and make coping more difficult.

Yusra Mardini, a prominent figure in the ROT, represents a high-profile example of resilience. She recounts towing a sinking inflatable boat across the Mediterranean to save 18 refugees, including her sister Sarah. Her narrative, marked by both survival and traumatic memories, illustrates the lasting psychological effects of displacement (Lewis, 2016).

“Maybe I'm going to die on the way, but I'm almost dead in my country. I can't do anything…

A few of the refugees could swim, so Yusra and her sister jumped into the sea. For the next three and a half hours, they dragged the broken-down boat toward the shore. *“The last half an hour, I could not manage anymore, so I got back into the boat. It was so cold. I look at the sea now, and I just feel faint.”* Mardini’s narrative demonstrates the power of sport to restore hope while simultaneously exposing the enduring psychological consequences of the refugee experience. Viewed through ACTM, Mardini’s story highlights severe transition barriers, but also the coping resources of athletic identity, survival meaning, and future-oriented hope that supported her adjustment.

Masomah Ali Zada, who served as EOR flagbearer and team leader, fled Taliban persecution in Afghanistan after facing harassment for cycling in “sporting gear” (InfoMigrants, 2021).

A lot of people felt it was wrong. Stopped us from threatening and insulting us, and threw stones at us.

Relocating to France, sport offered her opportunities to rebuild her life, yet family concerns remain. *“Even winning medals, I still fear for my family’s safety back home.”* Ali Zada’s journey demonstrates sport’s role in fostering social integration while underscoring the ongoing emotional challenges faced by refugee athletes separated from their families. In ACTM terms, her story illustrates that successful career transition does not eliminate barriers entirely; rather, coping remains an ongoing process shaped by safety concerns, transnational family ties, and emotional strain.

### 6.5 The another side of the coin: challenges faced by refugee team

Alongside the inspiring narratives of athletic achievement, it is critical to address the structural and psychosocial challenges encountered by the Refugee Olympic Team. One of the most significant pressures observed involves the socio-political context of host nations, as seen during the Paris 2024 Olympic Games. The athletes competed in a host country where the anti-immigrant far-right party saw a surge in voter support in the parliamentary elections, but was ultimately blocked by a coalition of the French left and fell short of a majority (International Rescue Committee, 2024). This situation reflects the broader impact of global mass migration on internal affairs and immigration policies, which are heavily influenced by societal reactions. Shifting political climates heighten psychosocial vulnerability, as anti-migrant rhetoric amplifies deportation fears and identity stress for refugee athletes (Scherer et al., 2024). While these athletes exemplify resilience and determination, their experiences highlight how these external stressors exacerbate anxiety and diminish wellbeing within precarious host support systems. ACTM is particularly useful here as it explains how strong barriers can undermine even well-supported transitions when the surrounding social environment becomes hostile or unstable.

The symbolic representation of refugee athletes has also been subject to critical scholarly scrutiny. According to Burdsey and colleagues, refugee athletes have often been politicised and celebrated as symbols of successful integration and hope for new immigrants in Europe (Burdsey et al., 2023). Yet, this temporary heroization rarely brings lasting benefits, as public support tends to fluctuate with political climates. Scheadler and Ledford (2018) demonstrate that the Refugee Olympic Team’s media impact remained limited, with negative political framings, such as the U.S. Travel Ban, proving more dominant. Ultimately, these athletes exist in a liminal space: they remain citizens of the nations they fled but are unable to represent them, and they also cannot compete for their host countries because they lack citizenship there. As a result, this team stands in contrast to the Olympic ideal of national unity, missing what Burdsey and colleagues describe as a crucial social transformation, the chance to gain citizenship in their new country and represent it through sport. This liminality can be read through Stambulova and Wylleman’s model as an unresolved transition state in which identity reconstruction remains incomplete because institutional and political resources are not sufficient to overcome structural barriers.

A recurring challenge is the perception that athletic success functions as a prerequisite for social acceptance. Burdsey (2020) characterises this as a “positive contrast” to so-called “flawed” immigrants who do not or cannot engage in socially, economically, or politically integrative activities like sport. However, this perception makes refugees’ social ‘value’ unstable and conditional, leaving refugees vulnerable to losing recognition and acceptance over time and exacerbating feelings of insecurity, anxiety and diminished wellbeing. ACTM clarifies that this conditional acceptance functions as a barrier, weakening coping and making belonging dependent on performance rather than on secure social inclusion. These dynamics set the stage for media representations explored next, where “model migrant” framing further complicates identity negotiation.

### 6.6 The representation of refugee Olympic team in the media

The media possesses the power to shape how information is presented and interpreted (Coakley, 2016). Consequently, it plays a crucial role in framing narratives surrounding the global refugee crisis (Bleiker et al., 2013). In a study examining media coverage of the Refugee Olympic Team during the Rio and Tokyo Olympic Games, it was found that the achievements of EOR athletes were warmly received, celebrated, and even glorified within both the sporting world and the media; however, the media largely failed to humanise their refugee status (Turcott and Ariyo, 2022). Media framings construct refugee athletes as “model migrants,” exceptional integration symbols, while obscuring everyday psychosocial realities like identity negotiation between origin/host loyalties and vulnerability to political rhetoric (Burdsey et al., 2023). From an ACTM perspective, this selective visibility is a double-edged resource: it can enhance belonging and hope, but it can also intensify pressure by tying legitimacy to success narratives. Moreover, some sociologists observed a shift in sentiment toward refugees within this process, moving from empathy to animosity (Georgiou and Zaborowski, 2017), with emphasis increasingly placed on issues such as migrant criminality, deportation, failed integration, and insecurity (Michelini, 2021a; Gutierrez Rodriguez, 2018). Taking into account the psychological trauma particularly experienced by forcibly displaced migrant women (Taknint et al., 2024), such framings intensify acculturative stress, where selective visibility fosters belonging yet heightens deportation anxiety amid shifting climates (Spaaij et al., 2014). The portrayal of EOR athletes as “model migrants” on the path to success has contributed to their recognition as exceptional individuals, while inadvertently reinforcing the perception that other refugees and migrants are a burden to society (Burdsey et al., 2023). Nonetheless, it can be argued that the Refugee Olympic Team has been effective in raising awareness and, through the stories of certain remarkable athletes, has the potential to mobilise international forums for solutions that offer hope to those in refugee situations and contribute to restoring their sense of dignity, belonging and psychosocial wellbeing (Nesson and Stoloff, 2016). ACTM suggests that this restorative potential depends on whether visibility is accompanied by durable support mechanisms rather than just symbolic admiration. Although the role of sport in promoting social inclusion has become widely acknowledged globally (International Olympic Committee (IOC), 2023), its effectiveness in transforming the exclusionary nature of the social structure needs to be measured concretely and through longitudinal studies (Spaaij et al., 2014; Suzuki, 2017). However, from a Bourdieusian perspective, it can be argued that migrants can contribute to the host society in social, cultural, and economic terms through their transnational ties, which function as various forms of capital that influence migrant psychosocial adaptation (Saksela-Bergholm et al., 2019).

Among the most prominent figures in the history of Olympic Refugee Teams is the Syrian swimmer Yusra Mardini. Mardini competed at both the 2016 Rio and the 2020 Tokyo Olympic Games, where she emerged as a humanitarian and sporting symbol of the refugee movement (Michelini, 2021b). Her story resonated strongly with the wider public, as her determination and resilience in the face of war and displacement led to her being heralded as a “symbol of hope” and established her as a leading figure within the Refugee Olympic Team (Blair, 2020). Furthermore, a biographical film titled “The Swimmers” was produced, which compellingly portrays the migration experience and conveys a message of hope within the refugee movement. The film narrates Mardini’s arduous journey as an Syrian war survivor and her evolving identity as an Olympic athlete, thereby contributing to social awareness of the humanitarian dimensions of the refugee experience (Ujayli, 2023). In addition, Mardini’s autobiography, “*Butterfly: From Refugee to Olympian - My Story of Rescue, Hope, and Triumph*” provides an in-depth personal account of her journey, further enriching both academic and public understanding of her experiences and the broader refugee narrative from a perspective that is closely connected to resilience, hope and psychological wellbeing (Mardini, 2018). These extended narratives illustrate the ACTM process over time: disruption, resource acquisition, coping, adaptation, and public identity reconstruction.

The Refugee Olympic Team initiative has also drawn criticism from the IOC and International Sports Federations (ISFs) for using refugee athletes as a form of tokenism or sportswashing. One reason for this criticism is that media coverage of refugee participation in the Olympics emphasises the IOC and UNHCR more than the refugees themselves. The representation of refugee athletes, particularly in media coverage of the Rio 2016 and Tokyo 2020 Olympic Games, is significantly lower than that of Thomas Bach and other IOC representatives (Kim, 2024b). It is also claimed that the IOC uses its discourse on refugees through the Refugee Olympic Team as a tool for authoritative self-representation and positive image creation to strengthen its hegemonic position in this field (Burdsey et al., 2023). Furthermore, while refugee athletes’ participation in the Games is presented as an inspirational and a success story, as Spaaij et al. (2019) noted, it can have the opposite effect by exposing participants to racial or religious discrimination. This criticism matters within ACTM because institutional visibility is not inherently supportive; when visibility serves institutional legitimacy more than athlete development, it can become another barrier to adjustment.

Several athletes within the Olympic Refugee Team have garnered significant media attention. Among them is Cindy Ngamba, a Cameroon-born, Britain-trained boxer who secured a historic bronze medal at the 2024 Paris Olympics and has been prominently featured in international coverage. Ngamba’s challenges as a refugee and her ascent in the sporting world are extensively documented in the documentary “Personal Best: Paris 2024” (Goh, 2025). Other notable athletes include Masomah Ali Zada, who served as the team’s sporting leader at Tokyo 2020 and has been highlighted in various media interviews advocating for refugee athletes (Peng, 2024; Batha, 2024), and Perina Lokure Nakang, a South Sudan-born athlete who competed at Paris 2024, sharing her refugee experience and athletic journey through international news features and interviews (Janetsky, 2024; Watta, 2023). Across these cases, ACTM helps explain why certain athletes become especially visible: they embody not only performance, but also narratives of coping, adaptation, and symbolic belonging.

It should be recalled that, in line with the changing social dynamics of our age, the Olympic Games have evolved into a structure that not only showcases athletes but is also recognised as a symbol representing universal human rights and global solidarity (Boykoff, 2016), thereby reflecting more inclusive, peaceful, and humanitarian values (Giffin et al., 2024). The symbolic call of the Olympics, traditionally expressed as “citius, altius, forties” (Golden, 2011), has, in the most recent edition of the Games hosted by Paris, been progressively reimagined, albeit unofficially, as “diversity, equality, and inclusivity” (Engin, 2025). Within the ACTM framework, these reimagining matters because they show that the Olympic environment can either facilitate or obstruct career transition, depending on whether inclusion is symbolic or substantively supported.

## 7 Conclusion

The Refugee Olympic Team represents more than a group of athletes participating in the Olympic Games; it reflects a significant transformation in the philosophy and practice of Olympism in the twenty-first century. By offering athletes who have experienced forced migration, trauma, and displacement the opportunity to compete on the global stage, the initiative creates a platform through which visibility, belonging, and psychosocial support may be fostered. For many displaced athletes, participation in the Olympic movement can help rebuild identity, restore dignity, and strengthen resilience through institutional and social support structures. In this sense, the Refugee Olympic Team symbolically mobilises the unifying potential of sport to promote peace, inclusion, and solidarity within the international sporting community while also increasing global awareness of forced migration. Read through the lens of the Athletic Career Transition Model (ACTM), these outcomes are not merely symbolic; they also represent transition processes shaped by demands, resources, barriers, and coping strategies that affect refugee athletes’ adjustment, wellbeing, and mental health.

This study set out to examine the Refugee Olympic Team as a psychologically meaningful and institutionally mediated career transition context, rather than as a purely descriptive humanitarian case. In line with that aim, the analysis integrated ACTM with the SDG-16 framework to examine how refugee athletes’ experiences of wellbeing, belonging, resilience, and adjustment are constructed across institutional and media discourse. The findings show that the ROT operates as a complex transition environment in which displaced athletes encounter both support and strain, visibility and vulnerability, recognition and conditional acceptance. Accordingly, the study foregrounds the psychological dimensions of refugee athletes’ experiences by embedding these dimensions within an explicit theoretical framework.

At the same time, the transformative potential of the Refugee Olympic Team for refugee wellbeing is shaped by how its narratives are constructed and circulated in public discourse. From a critical perspective, the limitations of this initiative become particularly evident in how refugee identities are framed in media and institutional narratives. Sport for Development critiques have highlighted how paternalistic hierarchies and forms of symbolic inclusion may be reproduced through tokenism and “model migrant” framing, thereby constraining athlete agency within broader structures of global sport governance. The category of “refugee” itself rarely carries positive social connotations. Instead, dominant narratives frequently emphasise exceptional individual success stories that portray members of the Refugee Olympic Team as inspirational or “miraculous” figures. While ostensibly celebratory, such narratives may reproduce a narrow stereotype that values only the most accomplished, well-integrated, and visibly successful refugees. While such representations may increase the global visibility for refugee athletes, they may also lead to the emergence of what scholars describe as the “model refugee” narrative. This framing can place implicit expectations on athletes to embody resilience, gratitude, and exceptional achievement, potentially creating psychosocial pressures related to identity representation and public perception. In this sense, media narratives do not merely reflect refugee experiences but actively shape the symbolic roles that refugee athletes are expected to perform within global sport discourse. The symbolic elevation of a limited number of highly visible athletes may therefore risk reinforcing a broader binary in which millions of other displaced individuals remain marginalised or invisible within public discourse. Consequently, institutional and media framings of the Refugee Olympic Team should be understood not only as celebratory representations but also as mechanisms that shape social perceptions of belonging, inclusion, and legitimacy within global migration debates. From an ACTM perspective, these same framings may function as both resources and barriers: they can enhance visibility and support, but they can also intensify pressure, conditional acceptance, and identity strain.

Taken together, the findings of this study highlight the dual character of the Refugee Olympic Team. On the one hand, the initiative functions as an important symbolic platform aligned with Sustainable Development Goal 16 by promoting visibility, inclusion, and psychosocial resilience among displaced athletes. On the other hand, its representation within media and institutional discourse reveals structural limitations related to selective visibility, symbolic inclusion, and the broader geopolitical dynamics that produce refugeehood. By critically examining these tensions, this study contributes to emerging scholarship at the intersection of sport, forced migration, and psychosocial wellbeing. More specifically, it contributes an ACTM-informed interpretation of ROT that shows how forced displacement, Olympic participation, and institutional representation intersect to shape athlete adjustment and long-term coping. Future research should prioritise empirical studies centred on the lived experiences of refugee athletes in order to deepen understanding of identity formation, belonging, and mental health within elite sport contexts shaped by displacement and global inequality. Such work would allow the psychological transition processes identified in this study to be tested more directly through interviews, longitudinal designs, and athlete-centred methods.

## Data Availability

The original contributions presented in this study are included in the article, and further inquiries can be directed to the corresponding author.
